# Metabolomics reveals distinct, antibody-independent, molecular signatures of MS, AQP4-antibody and MOG-antibody disease

**DOI:** 10.1186/s40478-017-0495-8

**Published:** 2017-12-06

**Authors:** Maciej Jurynczyk, Fay Probert, Tianrong Yeo, George Tackley, Tim D. W. Claridge, Ana Cavey, Mark R. Woodhall, Siddharth Arora, Torsten Winkler, Eric Schiffer, Angela Vincent, Gabriele DeLuca, Nicola R. Sibson, M. Isabel Leite, Patrick Waters, Daniel C. Anthony, Jacqueline Palace

**Affiliations:** 1 Nuffield Department of Clinical Neurosciences, John Radcliffe Hospital, University of Oxford, Level 3, West Wing, Headley Way, Oxford, OX3 9DU UK; 2 Department of Neurology, Medical University of Lodz, Lodz, Poland; 3 Department of Pharmacology, University of Oxford, Mansfield Road, Oxford, OX1 3QT UK; 4 Department of Neurology, National Neuroscience Institute, 11 Jalan Tan Tock Seng, Singapore, 308433 Singapore; 5 Chemistry Research Laboratory, Department of Chemistry, University of Oxford, Mansfield Road, Oxford, OX1 3TA UK; 6 Mathematical Institute, University of Oxford, Woodstock Rd, Oxford, OX2 6GC UK; 7 Numares AG, Am Biopark 9, 93053 Regensburg, Germany; 8 Cancer Research UK & Medical Research Council Oxford Institute for Radiation Oncology, Department of Oncology, University of Oxford, OX37DQ, Oxford, UK

**Keywords:** Multiple sclerosis, Neuromyelitis optica, Metabolomics, Biomarker, MOG antibody disease

## Abstract

The overlapping clinical features of relapsing remitting multiple sclerosis (RRMS), aquaporin-4 (AQP4)-antibody (Ab) neuromyelitis optica spectrum disorder (NMOSD), and myelin oligodendrocyte glycoprotein (MOG)-Ab disease mean that detection of disease specific serum antibodies is the gold standard in diagnostics. However, antibody levels are not prognostic and may become undetectable after treatment or during remission. Therefore, there is still a need to discover antibody-independent biomarkers. We sought to discover whether plasma metabolic profiling could provide biomarkers of these three diseases and explore if the metabolic differences are independent of antibody titre. Plasma samples from 108 patients (34 RRMS, 54 AQP4-Ab NMOSD, and 20 MOG-Ab disease) were analysed by nuclear magnetic resonance spectroscopy followed by lipoprotein profiling. Orthogonal partial-least squares discriminatory analysis (OPLS-DA) was used to identify significant differences in the plasma metabolite concentrations and produce models (mathematical algorithms) capable of identifying these diseases. In all instances, the models were highly discriminatory, with a distinct metabolite pattern identified for each disease. In addition, OPLS-DA identified AQP4-Ab NMOSD patient samples with low/undetectable antibody levels with an accuracy of 92%. The AQP4-Ab NMOSD metabolic profile was characterised by decreased levels of scyllo-inositol and small high density lipoprotein particles along with an increase in large low density lipoprotein particles relative to both RRMS and MOG-Ab disease. RRMS plasma exhibited increased histidine and glucose, along with decreased lactate, alanine, and large high density lipoproteins while MOG-Ab disease plasma was defined by increases in formate and leucine coupled with decreased myo-inositol. Despite overlap in clinical measures in these three diseases, the distinct plasma metabolic patterns support their distinct serological profiles and confirm that these conditions are indeed different at a molecular level. The metabolites identified provide a molecular signature of each condition which is independent of antibody titre and EDSS, with potential use for disease monitoring and diagnosis.

**Electronic supplementary material:**

The online version of this article (10.1186/s40478-017-0495-8) contains supplementary material, which is available to authorized users.

## Introduction

The field of central nervous system (CNS) inflammatory demyelinating diseases (IDD) has undergone considerable change with the discovery of antibodies against the aquaporin-4 water channel (AQP4-Ab) in neuromyelitis optica spectrum disorders (NMOSD) [30, 31]. More recently, antibodies against conformational epitopes of the myelin oligodendrocyte glycoprotein (MOG) have been reported in AQP4-Ab negative NMOSD [28, 35] as well as in pediatric acute disseminated encephalomyelitis (ADEM) [40]. The identification of these biomarkers, together with immunopathological studies, has led to their increasing recognition as distinct clinical entities separate from multiple sclerosis (MS) [25, 34, 46, 52, 53]. This has important prognostic and therapeutic implications, since it is now known that disability in AQP4-Ab NMOSD is wholly dependent on relapses and that MS-specific treatments are not effective in reducing relapses in these patients [29, 41, 55].

There has been controversy as to whether CNS IDD associated with MOG-Ab represents a distinct condition separate from MS. Early on, MOG was proposed as a candidate autoantigen for MS and MOG is still routinely used as an immunogen in animal models of MS [1, 6] including those used to explore the treatment mechanism of glatiramer acetate and fingolimod, both of which are approved drugs with proven efficacy in MS patients [10, 45]. In addition, the specificity of MOG-Abs remained a concern as MOG-Abs were found in patients with other inflammatory diseases and in healthy controls [24, 51]. Indeed, early studies revealed the presence of MOG-Ab in MS patients, however, these studies only detected antibodies against linear epitopes of MOG which were later found to not be clinically relevant [45]. Recent histopathology studies of patients with fully conformational MOG-Ab showed features compatible with pattern II MS pathology, reflecting humoral mediated mechanisms [42]. Observations of absent or very low levels of conformational MOG-Ab in MS patients, and reports of imaging features distinct from MS, supports that MOG-Ab disease is a separate clinical entity from both MS and AQP4-Ab NMOSD, although pathological biomarkers have not been explored up to now [21, 22, 42, 52].

MS is believed to be due to an aberrant T-cell response with B-cell mediated autoimmunity also playing a role [5], while autoantibodies are believed to be central to the pathogenesis of AQP4-Ab NMOSD [18], and MOG-Ab disease is now regarded as an antibody mediated condition. Despite these immunopathological differences, clinical features overlap which can make clinical distinction challenging [20]. RRMS, AQP4-Ab NMOSD, and MOG-Ab disease are all characterised by relapses which involve similar topographical regions within the CNS, interspersed with periods of remission. While several brain imaging studies have been able to distinguish MS from AQP4-Ab NMOSD or MOG-Ab disease, the almost identical presentation of the latter two conditions means distinction using radiological features alone is not possible [21, 22]. Thus, while the underlying mechanisms appear to be unique, the molecular processes which lead to convergent, downstream histological and radiological signs remain unknown. The absence of a biomarker for MS means that diagnosis is predicated on the exclusion of competing diagnoses and so, to date, reliable cell-based assays detecting antibodies against AQP4 and MOG remain the gold standard for diagnosing and differentiating these three conditions. In spite of this, the most sensitive assays for these antibodies are not widely available and can still fail to detect the antibodies in patients with low antibody titre when treated and / or outside of relapses.

Despite this invaluable role of AQP4 and MOG antibodies in diagnosis of CNS IDD, antibody titre does not appear to correlate with disease severity or predict relapses in the few published studies available [3, 17, 27, 50]. This is particularly true for AQP4 NMOSD, though in MOG-Ab disease this is less clear. MOG-Ab seems to rapidly decline following monophasic ADEM and persist in chronic CNS demyelinating conditions [44]. The consistent observation for both conditions, however, is that the antibody titre can decrease with treatment or when the disease is inactive, making the diagnosis in a small number of cases very problematic. Indeed, of the 54 AQP4-Ab NMOSD patients included in our study, 24 had low or undetectable antibody levels at the time of sample collection. Therefore, there is still a need for the discovery of antibody-independent biomarkers which would provide additional information on the molecular mechanisms underlying these diseases.

High-resolution ^1^H nuclear magnetic resonance (NMR) spectroscopy is a non-invasive tool, which, coupled with multivariate statistical analysis, identifies metabolite patterns in biofluid samples. We have previously shown that this technique is able to distinguish RRMS from healthy controls with 100% accuracy and from secondary progressive MS (SPMS) with an accuracy of 87% using the metabolite profile alone [7]. Here, using a similar approach, we present a comparison of the plasma metabolic profiles of patients with RRMS, AQP4-Ab NMOSD, and MOG-Ab disease, in an effort to uncover the metabolic signatures representative of each disease state. Their different metabolic profiles provide further evidence that these three diseases are indeed distinct, serving as potential diagnostic biomarkers which are independent of antibody levels and EDSS. The discriminatory metabolites identified also provide insight into the metabolic perturbations in each condition, allowing exploration of underlying pathophysiological mechanisms.

## Methods

### Patients

All patients included in the study were recruited from NMO and MS clinics in the John Radcliffe Hospital in Oxford. Patients were diagnosed with AQP4-Ab or MOG-Ab disease if they had at least one clinically evident inflammatory demyelinating event and tested positive on cell-based assays for AQP4-Ab or MOG-Ab, respectively [52, 53]. No patient was double positive for AQP4 and MOG Abs. AQP4-Ab end point titre was determined by the reciprocal of the highest positive serum dilution by cell-based assay. A fluorescence visual score of ≥1 (range 0–4) was used as the threshold for positivity. Patients were diagnosed with RRMS if they fulfilled the revised 2010 McDonald criteria [43]. All patients gave their written consent to participate in the study (Oxford Research Ethics Committee C Ref: 10/H0606/56 and 16/SC/0224). Patient demographics are shown in Table 1.

**Table 1:** Patient information

	RRMS	AQP4-Ab NMOSD	MOG-Ab
Number of patients	34	54	20
Age, mean (range), y	41 (18–60)	53 (22–83)*†	39 (16–70)
Gender, No. female (% female)	25 (74)	46 (85)	9 (45)
EDSS, median (range)	4 (0–7)	3 (0–8)	2 (0–8)
Disease duration, median (range), months	89 (1–301)	70 (3–270)	16 (1–420)*~
Time since relapse, median (range), months	23 (0–133)	22 (0–108)	8 (1–39)*~
On oral prednisolone, %	0%	85%	35%
On prednisolone, mean dose mg	0	12	14
On azathioprine, %	0	49	5
On methotrexate, %	0	11	5
On mycophenolate, %	0	19	5
On interferon β, %	15	0	5
On glatiramer, %	12	0	0
On fingolimod, %	12	0	0
On dimethyl fumarate, %	12	3	0
On natalizumab, %	15	0	0

The Kolmogorov-Smirnov test was used to identify significant differences of each class compared to RRMS (*), AQP4-Ab NMOSD (~), or MOG-Ab disease (†)

### Plasma sample collection

Blood was collected into vacutainer lithium-heparin tubes (Becton Dickinson, product number 367375) and stored at room temperature for 30 mins before centrifugation at 2200 x g for 10 mins. Plasma was immediately aliquoted and stored at −80 °C.

### NMR sample preparation

Plasma samples were defrosted at room temperature and centrifuged at 100,000 x g for 30 min at 4 °C. 150 μL of the plasma supernatant was then diluted with 450 μL of 75 mM sodium phosphate buffer prepared in D_2_O (pH 7.4). Samples were then centrifuged at 16,000 x g for 3 min to remove any precipitate before transferring to a 5-mm NMR tube.

### NMR spectroscopy

All NMR spectra were acquired using a 700-MHz Bruker AVII spectrometer operating at 16.4 T equipped with a ^1^H (^13^C/^15^N) TCI cryoprobe. Sample temperature was stable at 310 K. ^1^H NMR spectra were acquired using a 1D NOESY presaturation scheme for attenuation of the water resonance with a 2 s presaturation. A spin-echo Carr-Purcell-Meiboom-Gill (CPMG) sequence with a τ interval of 400 μs, 80 loops, 32 data collections, an acquisition time of 1.5 s, a relaxation delay of 2 s, and a fixed receiver gain was used to supress broad signals arising from large molecular weight plasma components. CPMG spectra provide a measurement of small molecular weight metabolites and mobile side chains of lipoproteins in the plasma sample and were used for all further analysis. Due to their large molecular weight, AQP4 and MOG-IgG antibody resonances are not observed in the ^1^H CPMG spectra. ^1^H correlation spectroscopy (COSY) spectra were acquired on at least one sample in each classification to aid in metabolite identification. For quality control, pooled plasma samples were spread throughout the run to monitor technical variation.

### NMR data preprocessing

Resulting free induction decays (FIDs) were zero-filled by a factor of 2 and multiplied by an exponential function corresponding to 0.30 Hz line broadening prior to Fourier transformation. All spectra were phased, baseline corrected (using a 3^rd^ degree polynomial), and chemical shifts referenced to the lactate-CH_3_ doublet resonance at δ = 1.33 ppm in Topspin 2.1 (Bruker, Germany). Spectra were visually examined for errors in baseline correction, referencing, spectral distortion, or contamination and then exported to ACD/Labs Spectrus Processor Academic Edition 12.01 (Advanced Chemistry Development, Inc.). The regions of the spectra between 0.08–4.20 ppm and 5.20–8.50 ppm were divided in to 0.02 ppm width ‘buckets’ and the absolute value of the integral of each spectral bucket was Pareto scaled. Resonances were assigned by reference to literature values [32, 47] and the Human Metabolome Database [56–58] and further confirmed by inspection of the 2D spectra, spiking of known compounds, and 1D–TOCSY spectra.

### Statistical analysis

The bucket integrals were imported into R software (R foundation for statistical computing, Vienna, Austria) [48]. All multivariate analysis was carried out using in-house R scripts and the *ropls* package [49]. Principal component analysis (PCA) was used to visualize the degree of separation between the disease classifications and detect potential outliers.

**Fig. 1:**
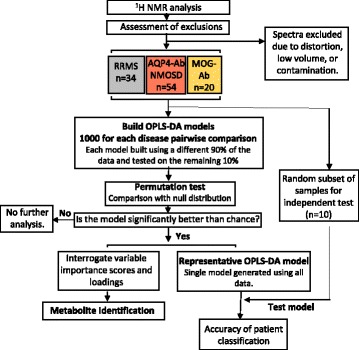
Illustration of the multivariate analysis methodology employed in this study

OPLS-DA was employed to investigate differences in the disease classifications using the scheme outlined in Fig. 1. The quality of classification was assessed by first correcting for unequal class sizes and then splitting the data into a training set (90%) used to build the model which is then tested on the remaining 10% of the data. This process was repeated to produce 1000 models in total. If these models perform better than models produced by random class assignments this ensures that the separation observed between the groups is valid. A final model is produced using all of the data and a completely independent set of samples (*n* = 10) is used to determine the predictive value of this model. A schematic of the statistical approach can be found in Additional file 1: Fig. S1. For a more detailed explanation of the analysis methods see the electronic Additional file 1.

### Lipoprotein subclass quantification

To interrogate the lipoprotein subclasses in more detail and obtain fully quantitative values the AXINON® lipoFIT® system (numares AG, Germany) was used. This test system deconvolutes the broad methyl lipoprotein resonance into its constituent parts allowing the direct measurement of the cholesterol content, number of particles, and mean particle diameter of each lipoprotein subpopulation. Lipoprotein groups measured include very low density lipoprotein (VLDL), low density lipoprotein (LDL), intermediate density lipoproteins (IDL), and high density lipoprotein (HDL) with each group further divided in to large and small subpopulations.

## Results

### Patients and potential confounders

NMR spectra from 108 patients (34 RRMS, 54 AQP4-Ab NMOSD, and 20 MOG-Ab disease) were included in this study (Fig. 1) to determine whether we could generate model algorithms that could accurately distinguish between plasma samples from patients with RRMS, AQP4-Ab NMOSD or MOG-Ab disease. Demographic and clinical data, including treatment regimes, for the patients in each group were collected (Table 1). No significant differences were observed in any of the parameters recorded between AQP4-Ab NMOSD samples with high titre (≥ 200) (*n* = 30) and low titre/negative samples (< 200) (*n* = 24).

Consistent with previous clinical reports, the majority of RRMS and AQP4-Ab patients were female (74% in the RRMS cohort and 85% in the AQP4-Ab NMOSD cohort) and the mean age of the AQP4-Ab NMOSD patients was higher (53 years in AQP4-Ab NMOSD compared to 41 and 39 in RRMS and MOG-Ab disease respectively) as a result of older age of onset in this condition. No significant differences or correlations were observed in any of the NMR data as a result of differences in the parameters described in Table 1, and multivariate analysis was unable to discriminate between the NMR spectra based on any of the factors recorded. Furthermore, the inclusion of gender, age, disease duration, time since relapse, or medication as variables in the multivariate analysis did not improve the OPLS-DA models, confirming that the differences in the parameters in Table 1 are not responsible for the discrimination between the three disease groups reported below. It was noted that the majority of AQP4-Ab NMOSD patients were on immunosuppression, whilst the majority of RRMS patients were on disease modifying therapies (DMTs). Therefore, the effect of each medication (listed in Table 1) on the metabolic profile was investigated in greater detail. In all cases, multivariate analysis was unable to discriminate patients on therapy versus those not on the therapy. For example, we were unable to build models to discriminate AQP4-Ab NMOSD plasma treated with steroids from those not treated with steroids. Importantly, RRMS and AQP4-Ab NMOSD patients not treated with steroids could still be distinguished from each other with high accuracy (Additional file 1: Fig. S2). This was the case for all medications listed in Table 1 including DMTs. Taken together, these findings suggest that none of the demographic and clinical parameters described in Table 1 had an appreciable effect on the metabolic profile.

### The NMR metabolite signature of RRMS plasma is distinct from AQP4-Ab NMOSD plasma

**Fig. 2:**
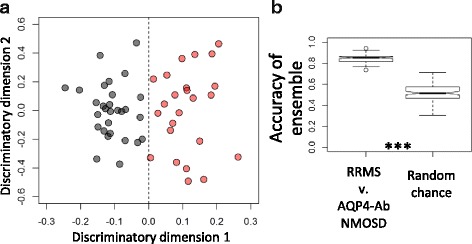
**a** OPLS-DA scores plot of RRMS (*black*) and AQP4-Ab NMOSD with titre ≥200 (*red*) NMR spectra. **b** OPLS-DA model validation. The accuracy of the ensemble of 1000 RRMS V. AQP4-Ab NMOSD models, as determined by classification of an independent test set, is significantly greater than that of random data. Kolmogorov-Smirnov test p-values <0.001 are represented by ***

**Fig. 3:**
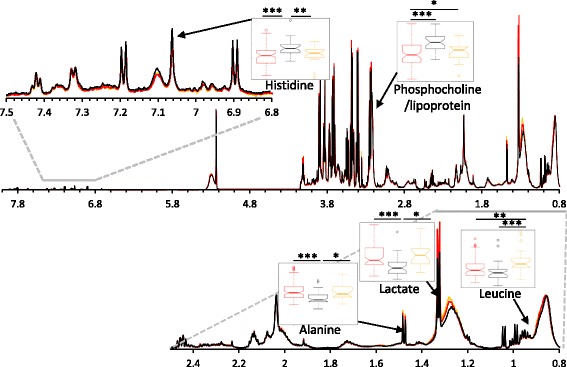
Average ^1^H CPMG spectra of RRMS (*black*), AQP4-Ab NMOSD (*red*), and MOG-Ab (yellow) plasma samples. Box plots illustrate significant differences in the NMR spectral integrals for a selection of metabolites selected by the OPLS-DA models. One-way ANOVA with post-hoc (Fisher’s LSD) *p*-values less than 0.05, 0.01, and 0.001 are represented by *, **, and *** respectively

Simultaneous measurement of multiple metabolites in plasma using ^1^H–NMR spectroscopy followed by the application of multivariate analysis (OPLS-DA) to generate predictive models (mathematical algorithms) was employed here to build models to discriminate between RRMS (*n* = 29) and AQP4-Ab NMOSD plasma with titre ≥200 (*n* = 25) at time of sampling. This subset was selected as it represents a diagnostically robust group of AQP4-Ab NMOSD patients suitable for model building. Inspection of the resulting OPLS-DA scores plot (Fig. 2a) revealed a significant difference in the NMR metabolic pattern of RRMS plasma compared to that of AQP4-Ab NMOSD patients with titre ≥200 at time of sampling (Fig. 3). Each point in the scores plot represents an NMR spectrum from a single patient while the axis represents variation in the metabolite profile; the metabolite pattern of points lying close together in the plot are similar. The distinct clusters observed for AQP4-Ab NMOSD or RRMS suggests the presence of a distinct metabolic profile for each condition. In order to confirm that this separation had not occurred by chance, and that the OPLS-DA model produced is predictive of RRMS/AQP4-Ab NMOSD status, a 10-fold cross validation scheme with repetition was employed as described in detail in the electronic Additional file 1. This approach validates the observed separation by creating an ensemble of OPLS-DA models from randomly selected (size-matched) subsets of the data. Each model was then tested on an independent subset of the samples (excluded from the training set) in order to establish the accuracy of the ensemble of models. The same approach was applied to a random set of data, produced by random class assignment of the NMR dataset, and the ensemble accuracies are compared. Figure 2b illustrates that the accuracy of the RRMS vs. APQ4-Ab NMOSD ensemble is significantly greater than that achieved by random chance and, in a rigorous manner, validates the separation observed and confirms that the metabolic signatures of these two diseases are distinct.

Investigation of the variables responsible for the separation in the models revealed several metabolites with significant perturbations. Scyllo-inositol, histidine, glucose, and a subset of small lipoprotein particles were significantly increased in RRMS plasma whilst lactate, alanine, and a subset of large lipoprotein particles were significantly decreased relative to AQP4-Ab NMOSD plasma.

**Fig. 4:**
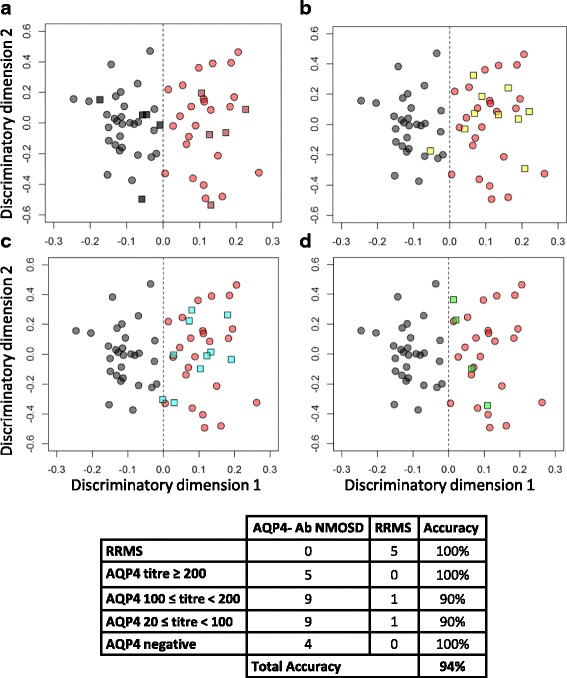
Predictive OPLS-DA model for the discrimination of RRMS and AQP4-Ab NMOSD plasma. OPLS-DA scores plots of RRMS (*black circle*) and AQP4-Ab NMOSD (*red circle*) and predicted classifications of **a**) independent test set of RRMS (black square) and AQP4-Ab NMOSD (red square), **b**) low titre (≥100 and <200) AQP4-Ab NMOSD (yellow square) **c**) very low titre (<100) AQP4-Ab NMOSD (*blue square*), and **d**) negative titre AQP4-Ab NMOSD (*green square*) plasma samples

As the above analysis proved that the discrimination between diseases did not occur by chance, it was valid to use the identified metabolites to predict disease classification. In order to further investigate the utility of the metabolites described and to produce a single algorithm for testing additional plasma samples, a single OPLS-DA model was produced using RRMS (*n* = 29) and high titre (≥200) AQP4-Ab NMOSD (*n* = 25) plasma NMR spectra validated above. This model was then tested with plasma samples from a randomly selected set of 10 entirely independent patients (5 with RRMS and 5 with AQP4-Ab NMOSD titres ≥200), i.e. these naïve samples were not used in the model validation at any stage. This final model correctly predicted the disease classes of this independent test set with an accuracy of 100% (Fig. 4a), confirming that the OPLS-DA model is able to identify AQP4-Ab NMOSD and RRMS using the NMR metabolite profile alone.

### The NMR metabolic profile identifies AQP4-Ab NMOSD and RRMS in a manner independent of antibody titre

We next investigated whether the metabolite profile could identify AQP4-Ab NMOSD patient samples with titre <200 or that were undetectable at the time of sampling (*n* = 24). All patients in this ‘low titre’ cohort had previously robust antibody titre results and a confirmed diagnosis of AQP4-Ab NMOSD. NMR spectra from AQP4-Ab NMOSD plasma with [100 ≤ titre <200] (Fig. 4b), [20 ≤ titre <100], (Fig. 4c), and negative results (Fig. 4d) at the time of sample collection were assessed using the OPLS-DA model generated above for AQP4-Ab NMOSD vs. RRMS. Of the 24 samples in this subset only two (one with a titre of 100 and one with a titre of 50) were incorrectly identified as RRMS by the OPLS-DA model, resulting in an accuracy of 92%. This finding indicates that the algorithm can identify AQP4-Ab NMOSD independent of antibody titre. Indeed, all of the AQP4-Ab NMOSD patients investigated whose antibody levels had become undetectable were correctly identified and no significant correlations or clustering on principle component analysis (Additional file 1: Fig. S3) were observed between any of the NMR metabolites measured and antibody titre. The same methods were used to investigate the effect of EDSS on the metabolite profile. We were unable to build models to discriminate between high and low EDSS patients and no clustering in the scores plots was observed (Additional file 1: Fig. S4). No significant differences were observed between the metabolite concentrations of the high and low AQP4-Ab titre samples and so these cohorts were combined into a single AQP4-Ab NMOSD (*n* = 54) cohort for all further analysis.

### The plasma NMR metabolic profile of MOG-Ab disease is distinct from both RRMS and AQP4-Ab NMOSD

**Fig. 5:**
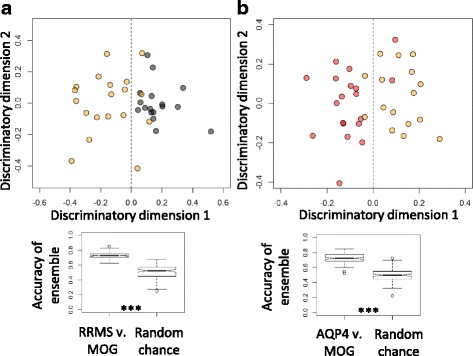
Statistically significant OPLS-DA models for the separation of MOG-Ab (*yellow*) from **a**) RRMS (*black*) and **b**) AQP4-Ab NMOSD (*red*). Model validity was assessed by comparing the accuracy, sensitivity, and specificity of the ensemble with that of a null distribution using the Kolmogorov-Smirnov test (p-values < 0.001 are indicated by ***)

OPLS-DA was able to discriminate between the plasma metabolic profiles of MOG-Ab disease and RRMS (Fig. 5a) and of MOG-Ab disease and AQP4-Ab NMOSD (Fig. 5b) with accuracies of 73 ± 4% and 73 ± 7% respectively. Once again, the accuracy of the ensemble of models was significantly greater than the null distribution (random chance models) validating the models and confirming that there are significant differences in the metabolic profiles of these three diseases. Interrogation of the discriminatory metabolites selected by the models revealed that the concentration of formate and leucine was increased in MOG-Ab plasma relative to both AQP4-Ab NMOSD and RRMS whilst the concentration of myo-inositol was decreased. A summary of the significant plasma metabolite changes is given in Table 2; a unique set of metabolites was found to vary significantly for each disease class relative to both other diseases illustrating that the plasma metabolite profiles of these three diseases are distinct. Quantitative titre information is not obtained from the MOG-Ab assay employed. Nevertheless, no correlation was observed between the metabolic profile and the semi-quantitative fluorescence visual score (Additional file 1: Fig. S5).

**Table 2:** Significant differences in the plasma metabolites of AQP4-Ab NMOSD, RRMS, and MOG-Ab disease

	AQP4-Ab NMOSD	RRMS	MOG-Ab
Concentration of large LDL particles	**↑**		
Size of HDL particles	**↑**		
Glucose	**↑**		
Cholesterol concentration in large HDL (subclass A)	**↑**		
Large LDL particles	**↑**		
Small HDL particles	**↓**		
Phosphocholine/lipoprotein	**↓**		
Scyllo-inositol	**↓**		
Lysine/creatinine/creatine		**↑**	
Histidine		**↑**	
Large HDL particles		**↓**	
Lactate		**↓**	
Unsaturated lipid		**↓**	
Alanine		**↓**	
Formate			**↑**
Leucine			**↑**
Myo-inositol			**↓**

Increases and decreases relative to the other two disease classifications are indicated with ↑ and ↓ respectively

Metabolites listed were identified as discriminatory by OPLS-DA

### Lipoprotein profiling reveals perturbations to plasma lipoprotein populations in AQP4-Ab NMOSD and RRMS

**Fig. 6:**
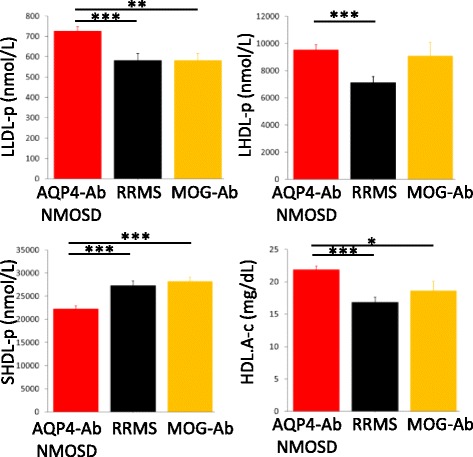
LipoFIT® variables with significant differences between groups AQP4-Ab NMOSD (*red*), RRMS (*black*), and MOG-Ab (*yellow*) following one-way ANOVA. Post-hoc (Fisher’s LSD) p-values less than 0.05, 0.01, and 0.001 are represented by *, **, and *** respectively. LLDL-p, large low density lipoprotein particle concentration; LHDL-p, large high density lipoprotein particle concentration; SHDL-p, small high density lipoprotein particle concentration; HDL.A-c, large high density lipoprotein cholesterol concentration

Lipoproteins were identified as highly discriminatory; the removal of lipoprotein measurements resulted in a marked decrease in model accuracy. However, the standard metabolomics NMR experiment (^1^H CPMG) is unable to categorize individual lipoprotein subpopulations, measure lipoprotein particle number, size, or cholesterol concentration. As a result, we investigated the plasma lipoproteins with an NMR-based lipidomics platform to define which lipoprotein subpopulations in RRMS plasma were different from those in AQP4-Ab NMOSD (Fig. 6). The number of large LDL and large HDL particles was significantly increased in AQP4-Ab NMOSD when compared with RRMS while the number of small HDL particles was significantly lower. As a consequence, an increase in the mean size of the HDL particles and an increase in the concentration of cholesterol in the subset of large HDL particles in AQP4-Ab NMOSD were also observed. Interestingly, no significant differences were observed in the total number of HDL particles or total HDL-cholesterol concentration between these diseases indicating that discrimination between these diseases would not be possible using a standard cholesterol test panel. All values obtained from this lipoFIT® analysis matched the intensities obtained from the CPMG NMR spectra further validating the significant differences in the metabolites identified by the multivariate analysis.

## Discussion

In this study, using ^1^H NMR spectroscopy, we demonstrate that RRMS, AQP4-Ab NMOSD, and MOG-Ab disease display distinct plasma metabolite patterns. In the case of AQP4-Ab NMOSD, the discriminatory metabolite pattern identified is independent of antibody titre. The most significant differences in metabolite concentrations observed across the three conditions included changes in plasma lipoprotein and amino acid levels along with changes in scyllo-inositol and myo-inositol. In particular, MOG-Ab disease which overlaps with AQP4-Ab NMOSD both clinically and in treatment response, was associated with unique changes in the levels of formate, leucine, and myo-inositol, allowing distinction from both RRMS and AQP4-Ab NMOSD. This separation between MOG-Ab disease and AQP4-Ab NMOSD has not been achieved by conventional MRI [21]. Thus, our observations contribute to the growing body of evidence that suggests that MOG-Ab disease represents a distinct clinical and pathophysiological entity. Since the metabolic profiles of MOG-Ab disease and AQP4-Ab NMOSD are different, despite them being predominantly humorally mediated conditions, their individual separation from RRMS is not simply a result of an up-regulated humoral immune response. Further investigation into the discriminatory metabolites could provide valuable information on the divergent pathophysiological processes underpinning each condition and their associated metabolic perturbations. However, the absence of direct histopathological correlates remains a limitation here as we cannot easily determine whether the magnitude of the responses is proportional to the CNS disease burden.

Different distributions of lipoprotein populations were observed in AQP4-Ab NMOSD and RRMS, small HDL was decreased and large LDL was increased in AQP4-Ab NMOSD relative to both RRMS and MOG-ab disease, while large HDL was decreased in RRMS relative to AQP4-Ab NMOSD. Total HDL and LDL particle number did not change significantly across the diseases suggesting that the lipoprotein subclasses have been modified while overall lipoprotein particle numbers remain the same. This is supported by the fact that no significant differences were observed in total cholesterol, LDL-cholesterol, HDL-cholesterol, or triglyceride concentrations and clarifies why a standard lipid panel is unable to discriminate between these three diseases. The lipoprotein population in AQP4-Ab NMOSD plasma is skewed towards larger particles while in RRMS plasma the lipoprotein particles are smaller. Our previous metabolomics study showed that RRMS serum has increased phosphocholine along with decreased β-hydroxybutyrate, and lipoprotein triacylglycerol (−CH_3_, −(CH_2_-)_n_, and –CH_2_CH_2_CO) concentrations with respect to SPMS patient serum [7]. In addition, a recent report demonstrated that RRMS serum LDL particles were smaller in RRMS compared to both SPMS and control samples [19], further supporting that lipoprotein populations are perturbed in MS. Indeed, altered lipid profiles have been previously linked with disease activity and progression in MS patients [54] although data on lipid alterations in non-MS CNS inflammatory diseases are lacking. In one of the few studies investigating lipoproteins in AQP4-Ab NMOSD patients, higher serum Apolipoprotein B levels were observed when compared with RRMS [33]. To the best of our knowledge, ours is the first study to compare plasma lipoprotein subclass data in RRMS, AQP4-Ab NMOSD, and MOG-Ab disease in detail.

The primary role of lipoprotein particles is the transport of lipid and other hydrophobic molecules around the body. Thus, lipoproteins are involved in a wide array of physiological processes including cell signaling, lipid homeostasis, and the acute phase response [8, 14, 23]. Previous studies have suggested that HDL particles are capable of crossing the blood brain barrier [12] and that LDL is present in the parenchyma of early MS lesions [39]. Lipoprotein modifications also occur in response to inflammation [2, 38]. Therefore, it is plausible that the plasma lipoprotein perturbations observed here are the result of lipoprotein modifications in response to CNS injury and the inflammatory response. Alternatively, changes in lipoproteins may reflect altered energy metabolism in these patients. Indeed, the plasma glucose concentration was higher in AQP4-Ab NMOSD and lactate decreased in RRMS plasma. Metabolomics analysis has previously revealed decreased lactate levels in cerebral spinal fluid of RRMS patients compared to AQP4-Ab NMOSD although no significant difference in glucose was observed in this biofluid [26].

Myo-inositol was previously reported to be low within spinal cord lesions in a group of 5 NMOSD patients (2 AQP4-Ab positive and 3 negative) [4] and elevated white matter myo-insoitol has been linked with multiple sclerosis [11]. Interestingly, the OPLS-DA model revealed that myo-inositol was higher in both AQP4-Ab NMOSD and RRMS relative to MOG-Ab disease. Serum scyllo-inositol concentration has been shown previously to be decreased in 73% of a heterogeneous population of NMOSD patients (both AQP4-Ab positive and negative) when compared with RRMS [37]. This is consistent with our observation that scyllo-inositol is decreased in AQP4-Ab NMOSD relative to both RRMS and MOG-Ab disease. Inositol phosphate and myo-inositol are components of myelin, and have roles in neural function and homeostasis in the CNS [13, 16, 36] and so the plasma myo-inositol and scyllo-inositol concentrations may reflect perturbations in inositol phosphate metabolism as a result of demyelination. Taken together, the set of metabolic perturbations identified may reflect alterations in lipid transport, membrane breakdown, and energy metabolism.

The data presented demonstrates that, in the case of AQP4-Ab NMOSD, the metabolite profile is independent of antibody titre. The OPLS-DA model was able to correctly identify AQP4-Ab NMOSD samples with antibody titres <200 at the time of sampling with an accuracy of 92%, whilst high titre (≥ 200) AQP4-Ab NMOSD samples and RRMS samples were discriminated with 100% accuracy. This finding suggests that the metabolic profile could be diagnostically useful in cases of suspected AQP4-Ab NMOSD where antibody level has decreased or become undetectable over time with treatment. Patients in the acute setting are often treated empirically and the rarer diagnosis of NMOSD is usually addressed downstream. Thus, metabolic profiling may be particularly useful in cases where samples are taken after established immunosuppression and / or outside of onset of relapse. Whilst this may only be the case in a small number of individuals, we believe it is particularly noteworthy as, out of the 54 AQP4-Ab NMOSD patients (randomly sampled from the Oxford NMO clinic) included in this study, 7% (*n* = 4) were seronegative at the time of sampling. Nevertheless, the samples from these four patients were correctly identified as AQP4-Ab NMOSD by the OPLS-DA model. We explored whether this observation is also true of MOG-Ab disease. However, due to the lower incidence of this condition, only two samples from patients who were seronegative at time of sampling were available for analysis. Whilst both of these samples were correctly identified as MOG-Ab disease by the OPLS-DA model (data not shown), future work on a larger cohort will confirm whether the metabolic profile is independent of MOG-Ab serostatus at sampling time-point. Given that the current work suggests that the AQP4-Ab NMOSD metabolic profile is independent of antibody titre, and that observational studies have reported that titres are not predictive of relapses, future work will explore the role of the discriminatory metabolites as biomarkers of disease activity.

Differences were observed in some of the patient demographics between disease groups which were consistent with previous clinical reports and are in keeping with known differences in the demographics of these conditions. Most notably, significant differences in medication and gender ratio were observed. All potential confounding factors were assessed in detail and none of the parameters listed in Table 1 were found to have a significant impact on the metabolite concentrations measured. Addition of these parameters as variables in the OPLS-DA model did not improve accuracy confirming that the separation observed between the diseases was not due to any differences in patient demographics. In particular, we investigated the effect of steroid treatment on the metabolic profile due to the high proportion of AQP4-Ab NMOSD and MOG-Ab disease patients treated with steroids compared to RRMS. OPLS-DA was unable to discriminate between steroid-treated and steroid-untreated AQP4-Ab NMOSD patients, whilst the models separating AQP4-Ab NMOSD without steroid treatment and RRMS patients (none of which were on steroids) were still predictive. Steroid treatment is known to alter the lipoprotein profile, with the primary effect of prednisolone being an increase in total LDL cholesterol concentration [9]. However, total LDL cholesterol concentration was not found to be significantly altered between the AQP4-Ab/RRMS/MOG-Ab samples tested. The same approach was used for every treatment (including DMTs) listed in Table 1 and no treatment had a significant effect on the strength of the models. We also demonstrated that EDSS does not impact the metabolite profiles of these three diseases and does not drive the discriminatory models produced (Additional file 1: Fig. S4) confirming that the biomarkers identified are independent of EDSS and instead represent the underlying pathology.

In conclusion, in this study we report unique plasma metabolite profiles in RRMS, AQP4-Ab NMOSD and MOG-Ab disease, and demonstrate that they differ significantly from each other, allowing for a highly predictive discrimination of these three diseases. The plasma metabolites underlying the separations were linked with lipoprotein populations, amino acids, glucose, lactate, formate, myo-inositol, and scyllo-inositol and may reflect metabolite pathway changes induced by demyelination, inflammation, blood brain barrier breakdown, and disruptions to energy metabolism. Our findings also show that the plasma NMR metabolic profile is capable of discriminating AQP4-Ab NMOSD from RRMS independently of antibody titre. Future work will examine whether the models developed in this study can be applied to childhood forms of demyelinating disease (in which MOG-Abs are commonly detected [15]), aid classification of heterogeneous cohorts such as antibody-negative NMOSD and atypical MS, and whether the discriminatory metabolites can be utilized as biomarkers for disease activity.

## Additional file

Additional file 1: Detailed description of statistical methods, supplementary OPLS-DA scores plots, and box plots of discriminatory metabolites. (PDF 1136 kb)

## References

[1] Anthony DC, Dickens AM, Seneca N, Couch Y, Campbell S, Checa B, Kersemans V, Warren EA, Tredwell M, Sibson NR, Gouverneur V, Leppert D (2014). Anti-CD20 inhibits T cell-mediated pathology and microgliosis in the rat brain. Ann Clin Transl Neurol.

[2] Barter P (2005). The inflammation: lipoprotein cycle. Atheroscler Suppl.

[3] Chanson JB, Alame M, Collongues N, Blanc F, Fleury M, Rudolf G, de Seze J, Vincent T (2013) Evaluation of clinical interest of anti-aquaporin-4 autoantibody followup in neuromyelitis optica. Clin Dev Immunol 2013:146219. doi:10.1155/2013/146219 10.1155/2013/146219 PMC3655457 23710199

[4] Ciccarelli O, Thomas DL, De Vita E, Wheeler-Kingshott CA, Kachramanoglou C, Kapoor R, Leary S, Matthews L, Palace J, Chard D, Miller DH, Toosy AT, Thompson AJ (2013). Low myo-inositol indicating astrocytic damage in a case series of neuromyelitis optica. Ann Neurol.

[5] Dendrou CA, Fugger L, Friese MA (2015). Immunopathology of multiple sclerosis. Nat Rev Immunol.

[6] Dickens AM, Larkin JR, Davis BG, Griffin JL, Claridge TD, Sibson NR, Anthony DC (2015). NMR-based metabolomics separates the distinct stages of disease in a chronic relapsing model of multiple sclerosis. J NeuroImmune Pharmacol.

[7] Dickens AM, Larkin JR, Griffin JL, Cavey A, Matthews L, Turner MR, Wilcock GK, Davis BG, Claridge TD, Palace J, Anthony DC, Sibson NR (2014). A type 2 biomarker separates relapsing-remitting from secondary progressive multiple sclerosis. Neurology.

[8] Dobreva I, Waeber G, Widmann C (2006). Lipoproteins and mitogen-activated protein kinase signaling: a role in atherogenesis. Curr Opin Lipidol.

[9] Ettinger WH, Hazzard WR (1988). Prednisone increases very low density lipoprotein and high density lipoprotein in healthy men. Metabolism.

[10] Farooqi N, Gran B, Constantinescu CS (2010). Are current disease-modifying therapeutics in multiple sclerosis justified on the basis of studies in experimental autoimmune encephalomyelitis?. J Neurochem.

[11] Fernando KT, McLean MA, Chard DT, MacManus DG, Dalton CM, Miszkiel KA, Gordon RM, Plant GT, Thompson AJ, Miller DH (2004). Elevated white matter myo-inositol in clinically isolated syndromes suggestive of multiple sclerosis. Brain.

[12] Ferretti G, Bacchetti T (2011). Peroxidation of lipoproteins in multiple sclerosis. J Neurol Sci.

[13] Fisher SK, Novak JE, Agranoff BW (2002). Inositol and higher inositol phosphates in neural tissues: homeostasis, metabolism and functional significance. J Neurochem.

[14] Ginsberg HN (1998). Lipoprotein physiology. Endocrinol Metab Clin N Am.

[15] Hacohen Y, Mankad K, Chong WK, Barkhof F, Vincent A, Lim M, Wassmer E, Ciccarelli O, Hemingway C (2017). Diagnostic algorithm for relapsing acquired demyelinating syndromes in children. Neurology.

[16] Haris M, Cai K, Singh A, Hariharan H, Reddy R (2011). In vivo mapping of brain myo-inositol. NeuroImage.

[17] Jarius S, Aboul-Enein F, Waters P, Kuenz B, Hauser A, Berger T, Lang W, Reindl M, Vincent A, Kristoferitsch W (2008). Antibody to aquaporin-4 in the long-term course of neuromyelitis optica. Brain.

[18] Jarius S, Wildemann B (2010). AQP4 antibodies in neuromyelitis optica: diagnostic and pathogenetic relevance. Nat Rev Neurol.

[19] Jorissen W, Wouters E, Bogie JF, Vanmierlo T, Noben JP, Sviridov D, Hellings N, Somers V, Valcke R, Vanwijmeersch B, Stinissen P, Mulder MT, Remaley AT, Hendriks JJ (2017). Relapsing-remitting multiple sclerosis patients display an altered lipoprotein profile with dysfunctional HDL. Sci Rep.

[20] Jurynczyk M, Craner M, Palace J (2015). Overlapping CNS inflammatory diseases: differentiating features of NMO and MS. J Neurol Neurosurg Psychiatry.

[21] Jurynczyk M, Geraldes R, Probert F, Woodhall MR, Waters P, Tackley G, DeLuca G, Chandratre S, Leite MI, Vincent A, Palace J (2017). Distinct brain imaging characteristics of autoantibody-mediated CNS conditions and multiple sclerosis. Brain.

[22] Jurynczyk M, Tackley G, Kong Y, Geraldes R, Matthews L, Woodhall M, Waters P, Kuker W, Craner M, Weir A, DeLuca GC, Kremer S, Leite MI, Vincent A, Jacob A, de Seze J, Palace J (2017). Brain lesion distribution criteria distinguish MS from AQP4-antibody NMOSD and MOG-antibody disease. J Neurol Neurosurg Psychiatry.

[23] Kaji H (2013). High-density lipoproteins and the immune system. J Lipids.

[24] Karni A, Bakimer-Kleiner R, Abramsky O, Ben-Nun A (1999). Elevated levels of antibody to myelin oligodendrocyte glycoprotein is not specific for patients with multiple sclerosis. Arch Neurol.

[25] Ketelslegers IA, Van Pelt DE, Bryde S, Neuteboom RF, Catsman-Berrevoets CE, Hamann D, Hintzen RQ (2015). Anti-MOG antibodies plead against MS diagnosis in an acquired demyelinating syndromes cohort. Mult Scler.

[26] Kim HH, Jeong IH, Hyun JS, Kong BS, Kim HJ, Park SJ (2017). Metabolomic profiling of CSF in multiple sclerosis and neuromyelitis optica spectrum disorder by nuclear magnetic resonance. PLoS One.

[27] Kitley J, Woodhall M, Leite MI, Palace J, Vincent A, Waters P (2015). Aquaporin-4 antibody isoform binding specificities do not explain clinical variations in NMO. Neurol Neuroimmunol Neuroinflamm.

[28] Kitley J, Woodhall M, Waters P, Leite MI, Devenney E, Craig J, Palace J, Vincent A (2012). Myelin-oligodendrocyte glycoprotein antibodies in adults with a neuromyelitis optica phenotype. Neurology.

[29] Kleiter I, Hellwig K, Berthele A, Kumpfel T, Linker RA, Hartung HP, Paul F, Aktas O, Neuromyelitis Optica Study G (2012). Failure of natalizumab to prevent relapses in neuromyelitis optica. Arch Neurol.

[30] Lennon VA, Kryzer TJ, Pittock SJ, Verkman AS, Hinson SR (2005). IgG marker of optic-spinal multiple sclerosis binds to the aquaporin-4 water channel. J Exp Med.

[31] Lennon VA, Wingerchuk DM, Kryzer TJ, Pittock SJ, Lucchinetti CF, Fujihara K, Nakashima I, Weinshenker BG (2004). A serum autoantibody marker of neuromyelitis optica: distinction from multiple sclerosis. Lancet.

[32] Lenz EM, Bright J, Wilson ID, Morgan SR, Nash AF (2003). A 1H NMR-based metabonomic study of urine and plasma samples obtained from healthy human subjects. J Pharm Biomed Anal.

[33] Li Y, Wang H, Hu X, Peng F, Yang Y (2010). Serum lipoprotein levels in patients with neuromyelitis optica elevated but had little correlation with clinical presentations. Clin Neurol Neurosurg.

[34] Lucchinetti CF, Guo Y, Popescu BF, Fujihara K, Itoyama Y, Misu T (2014). The pathology of an autoimmune astrocytopathy: lessons learned from neuromyelitis optica. Brain Pathol.

[35] Mader S, Gredler V, Schanda K, Rostasy K, Dujmovic I, Pfaller K, Lutterotti A, Jarius S, Di Pauli F, Kuenz B, Ehling R, Hegen H, Deisenhammer F, Aboul-Enein F, Storch MK, Koson P, Drulovic J, Kristoferitsch W, Berger T, Reindl M (2011). Complement activating antibodies to myelin oligodendrocyte glycoprotein in neuromyelitis optica and related disorders. J Neuroinflammation.

[36] Morell PQ,RH (1999). Characteristic Compostion of myelin. In: basic neurochemistry: molecular, cellular adn medical aspects. 6th edition.

[37] Moussallieh FM, Elbayed K, Chanson JB, Rudolf G, Piotto M, De Seze J, Namer IJ (2014). Serum analysis by 1H nuclear magnetic resonance spectroscopy: a new tool for distinguishing neuromyelitis optica from multiple sclerosis. Mult Scler.

[38] Navab M, Anantharamaiah GM, Fogelman AM (2005). The role of high-density lipoprotein in inflammation. Trends Cardiovasc Med.

[39] Newcombe J, Li H, Cuzner ML (1994). Low density lipoprotein uptake by macrophages in multiple sclerosis plaques: implications for pathogenesis. Neuropathol Appl Neurobiol.

[40] O'Connor KC, McLaughlin KA, De Jager PL, Chitnis T, Bettelli E, Xu C, Robinson WH, Cherry SV, Bar-Or A, Banwell B, Fukaura H, Fukazawa T, Tenembaum S, Wong SJ, Tavakoli NP, Idrissova Z, Viglietta V, Rostasy K, Pohl D, Dale RC, Freedman M, Steinman L, Buckle GJ, Kuchroo VK, Hafler DA, Wucherpfennig KW (2007). Self-antigen tetramers discriminate between myelin autoantibodies to native or denatured protein. Nat Med.

[41] Palace J, Leite MI, Nairne A, Vincent A (2010). Interferon Beta treatment in neuromyelitis optica: increase in relapses and aquaporin 4 antibody titers. Arch Neurol.

[42] Peschl P, Bradl M, Hoftberger R, Berger T, Reindl M (2017). Myelin Oligodendrocyte glycoprotein: deciphering a target in inflammatory demyelinating diseases. Front Immunol.

[43] Polman CH, Reingold SC, Banwell B, Clanet M, Cohen JA, Filippi M, Fujihara K, Havrdova E, Hutchinson M, Kappos L, Lublin FD, Montalban X, O'Connor P, Sandberg-Wollheim M, Thompson AJ, Waubant E, Weinshenker B, Wolinsky JS (2011). Diagnostic criteria for multiple sclerosis: 2010 revisions to the McDonald criteria. Ann Neurol.

[44] Probstel AK, Dornmair K, Bittner R, Sperl P, Jenne D, Magalhaes S, Villalobos A, Breithaupt C, Weissert R, Jacob U, Krumbholz M, Kuempfel T, Blaschek A, Stark W, Gartner J, Pohl D, Rostasy K, Weber F, Forne I, Khademi M, Olsson T, Brilot F, Tantsis E, Dale RC, Wekerle H, Hohlfeld R, Banwell B, Bar-Or A, Meinl E, Derfuss T (2011). Antibodies to MOG are transient in childhood acute disseminated encephalomyelitis. Neurology.

[45] Ramanathan S, Dale RC, Brilot F (2016). Anti-MOG antibody: the history, clinical phenotype, and pathogenicity of a serum biomarker for demyelination. Autoimmun Rev.

[46] Sato DK, Callegaro D, Lana-Peixoto MA, Waters PJ, de Haidar Jorge FM, Takahashi T, Nakashima I, Apostolos-Pereira SL, Talim N, Simm RF, Lino AM, Misu T, Leite MI, Aoki M, Fujihara K (2014). Distinction between MOG antibody-positive and AQP4 antibody-positive NMO spectrum disorders. Neurology.

[47] Tang H, Wang Y, Nicholson JK, Lindon JC (2004). Use of relaxation-edited one-dimensional and two dimensional nuclear magnetic resonance spectroscopy to improve detection of small metabolites in blood plasma. Anal Biochem.

[48] Team RC R: A Language and Environment for Statistical Computing. R Found Stat Comput

[49] Thevenot EA, Roux A, Xu Y, Ezan E, Junot C (2015). Analysis of the human adult urinary Metabolome variations with age, body mass index, and gender by implementing a comprehensive workflow for Univariate and OPLS statistical analyses. J Proteome Res.

[50] Valentino P, Marnetto F, Granieri L, Capobianco M, Bertolotto A (2017). Aquaporin-4 antibody titration in NMO patients treated with rituximab: a retrospective study. Neurol Neuroimmunol Neuroinflamm.

[51] Wang H, Munger KL, Reindl M, O'Reilly EJ, Levin LI, Berger T, Ascherio A (2008). Myelin oligodendrocyte glycoprotein antibodies and multiple sclerosis in healthy young adults. Neurology.

[52] Waters P, Woodhall M, O'Connor KC, Reindl M, Lang B, Sato DK, Jurynczyk M, Tackley G, Rocha J, Takahashi T, Misu T, Nakashima I, Palace J, Fujihara K, Leite MI, Vincent A (2015). MOG cell-based assay detects non-MS patients with inflammatory neurologic disease. Neurol Neuroimmunol Neuroinflamm.

[53] Waters PJ, McKeon A, Leite MI, Rajasekharan S, Lennon VA, Villalobos A, Palace J, Mandrekar JN, Vincent A, Bar-Or A, Pittock SJ (2012). Serologic diagnosis of NMO: a multicenter comparison of aquaporin-4-IgG assays. Neurology.

[54] Weinstock-Guttman B, Zivadinov R, Horakova D, Havrdova E, Qu J, Shyh G, Lakota E, O'Connor K, Badgett D, Tamano-Blanco M, Tyblova M, Hussein S, Bergsland N, Willis L, Krasensky J, Vaneckova M, Seidl Z, Ramanathan M (2013). Lipid profiles are associated with lesion formation over 24 months in interferon-beta treated patients following the first demyelinating event. J Neurol Neurosurg Psychiatry.

[55] Wingerchuk DM, Pittock SJ, Lucchinetti CF, Lennon VA, Weinshenker BG (2007). A secondary progressive clinical course is uncommon in neuromyelitis optica. Neurology.

[56] Wishart DS, Jewison T, Guo AC, Wilson M, Knox C, Liu Y, Djoumbou Y, Mandal R, Aziat F, Dong E, Bouatra S, Sinelnikov I, Arndt D, Xia J, Liu P, Yallou F, Bjorndahl T, Perez-Pineiro R, Eisner R, Allen F, Neveu V, Greiner R, Scalbert A (2013). HMDB 3.0--the human Metabolome database in 2013. Nucleic Acids Res.

[57] Wishart DS, Knox C, Guo AC, Eisner R, Young N, Gautam B, Hau DD, Psychogios N, Dong E, Bouatra S, Mandal R, Sinelnikov I, Xia J, Jia L, Cruz JA, Lim E, Sobsey CA, Shrivastava S, Huang P, Liu P, Fang L, Peng J, Fradette R, Cheng D, Tzur D, Clements M, Lewis A, De Souza A, Zuniga A, Dawe M, Xiong Y, Clive D, Greiner R, Nazyrova A, Shaykhutdinov R, Li L, Vogel HJ, Forsythe I (2009). HMDB: a knowledgebase for the human metabolome. Nucleic Acids Res.

[58] Wishart DS, Tzur D, Knox C, Eisner R, Guo AC, Young N, Cheng D, Jewell K, Arndt D, Sawhney S, Fung C, Nikolai L, Lewis M, Coutouly MA, Forsythe I, Tang P, Shrivastava S, Jeroncic K, Stothard P, Amegbey G, Block D, Hau DD, Wagner J, Miniaci J, Clements M, Gebremedhin M, Guo N, Zhang Y, Duggan GE, Macinnis GD, Weljie AM, Dowlatabadi R, Bamforth F, Clive D, Greiner R, Li L, Marrie T, Sykes BD, Vogel HJ, Querengesser L (2007). HMDB: the human Metabolome database. Nucleic Acids Res.

