# Supplementary material for: Metabolomics reveals distinct, antibody-independent, molecular signatures of MS, AQP4-antibody and MOG-antibody disease
Source: Acta Neuropathol Commun. 2017 Dec 6;5:95. doi: 10.1186/s40478-017-0495-8 (PMC5718082; doi:10.1186/s40478-017-0495-8)
Supplement: Additional file 1: — Detailed description of statistical methods, supplementary OPLS-DA scores plots, and box plots of discriminatory metabolites. (PDF 1136 kb) [file 40478_2017_495_MOESM1_ESM.pdf]

Supplementary Information

# Metabolomics reveals distinct, antibody-independent, molecular signatures of MS, AQP4-antibody and MOG-antibody disease.

Maciej Jurynczyk, MD, PhD*[1,2], Fay Probert, PhD*[3], Tianrong Yeo, MRCP[3,4], George Tackley, MD[1], Tim D.W. Claridge, DPhil[5], Ana Cavey[1], Mark R. Woodhall, PhD[1], Siddharth Arora, DPhil[6], Torsten Winkler, PhD[7], Eric Schiffer, PhD[7], Angela Vincent, FRCPath[1], Gabriele DeLuca, MD, DPhil[1], Nicola R. Sibson, PhD[8], M. Isabel Leite, DPhil[1], Patrick Waters, PhD[1], Daniel C Anthony, PhD**[3], and Jacqueline Palace, DM**[1].

***these authors contributed equally**

****these authors contributed equally**

1. Nuffield Department of Clinical Neurosciences, Level 3, West Wing, John Radcliffe Hospital, University of Oxford, Headley Way, Oxford OX3 9DU, UK.
2. Department of Neurology, Medical University of Lodz, Lodz, Poland.
3. Department of Pharmacology, University of Oxford, Mansfield Road, Oxford OX1 3QT, UK.
4. Department of Neurology, National Neuroscience Institute, 11 Jalan Tan Tock Seng, Singapore 308433, Singapore
5. Chemistry Research Laboratory, Department of Chemistry, University of Oxford, Mansfield Road, Oxford OX1 3TA, UK.
6. Mathematical Institute, University of Oxford, Woodstock Rd., Oxford, OX2 6GC, UK.
7. numares AG, Am Biopark 9, 93053 Regensburg, Germany.
8. Cancer Research UK & Medical Research Council Oxford Institute for Radiation Oncology, Department of Oncology, University of Oxford, Oxford, OX37DQ, UK.

**Correspondence to:** Dr Jacqueline Palace (clinical neurology), Neurosciences Offices, Level 3, West Wing, John Radcliffe Hospital, Headley Way, OX3 9DU Oxford, Tel: 01865 231900, Email: jacqueline.palace@ndcn.ox.ac.uk; or Dr Fay Probert (NMR spectroscopy/technical aspects/statistics), Department of Pharmacology, Mansfield Road, Oxford, OX1 3QT, Tel: +44(0)1865 281135, fay.probert@pharm.ox.ac.uk

**Key words:** Multiple sclerosis, neuromyelitis optica, metabolomics, biomarker, MOG antibody disease

## Detailed statistical methods.

Stage 1. Model validation

OPLS-DA was employed to investigate differences in the disease classifications using the schemes outlined in Fig. 1 and Fig. 2. OPLS-DA models were optimized by internal 7-fold cross-validation. The quality of classification was assessed using a 10-fold external cross-validation scheme with 1000 repetitions in total, correcting for unequal class sizes. This validation scheme involves multiple iterations of splitting the data into training and testing sets, which ensures that any discrimination observed in the models cannot have occurred by chance. The training data is used to estimate the model parameters and learn the underlying discriminatory patterns between the groups under consideration, whereas the independent test set is employed to assess the accuracy and generalizability of the trained models in the ensemble (Fig. 1). We quantified the outcome of the cross validation by calculating the accuracy, sensitivity, and specificity of each model (n = 1000) from the predicted classifications of the external independent test set, which was not used to build each model. It is important to appreciate that the classifier (OPLS-DA) was blinded to each test set when training each model. This validation scheme tends to avoid over-fitting and helps assess the generalizability of the model to previously unseen datasets. For an exhaustive discussion on validation of this approach see Arlot and Celisse (2010). These values were compared with those of a null distribution (obtained from randomly permuting the classifications) using the two-sided Kolmogorov-Smirnov test (significant if p-value 0.05 or less).

Stage 2. Prediction and identification of discriminatory metabolites.

If stage 1 of the analysis reveals that the OPLS-DA classifier performs significantly better than chance, the separation observed between classes is confirmed and it is valid to interrogate metabolites driving the separation and to use the data for prediction of additional samples. Discriminatory variables were identified by calculating the average of the variable

importance (VIP) scores of the ensemble of models. A VIP cut-off of 1.5 was used to identify the most important variables driving the separation between classes and, hence, the metabolites with significant differences between the diseases. The fold changes of these variables were further investigated by analysis-of-variance (ANOVA) followed by Tukey's honest significant difference (HSD) post-hoc test. The p-values obtained were then corrected for multiple comparisons using the Bonferroni correction. To assess if the OPLS-DA was able to identify AQP4-Ab NMOSD plasma with low/negative antibody levels at the time of sampling, a single predictive model was trained using the RRMS (n = 29)_and high titre (≥200) AQP4-Ab NMOSD (n = 25) plasma spectra (following successful validation using the method described in stage 1). This predictive model was subsequently tested using a completely independent naïve set of RRMS (n = 5) and AQP4-Ab NMOSD (n = 5) plasma samples from additional patients. These samples were not used in the model validation (stage 1).

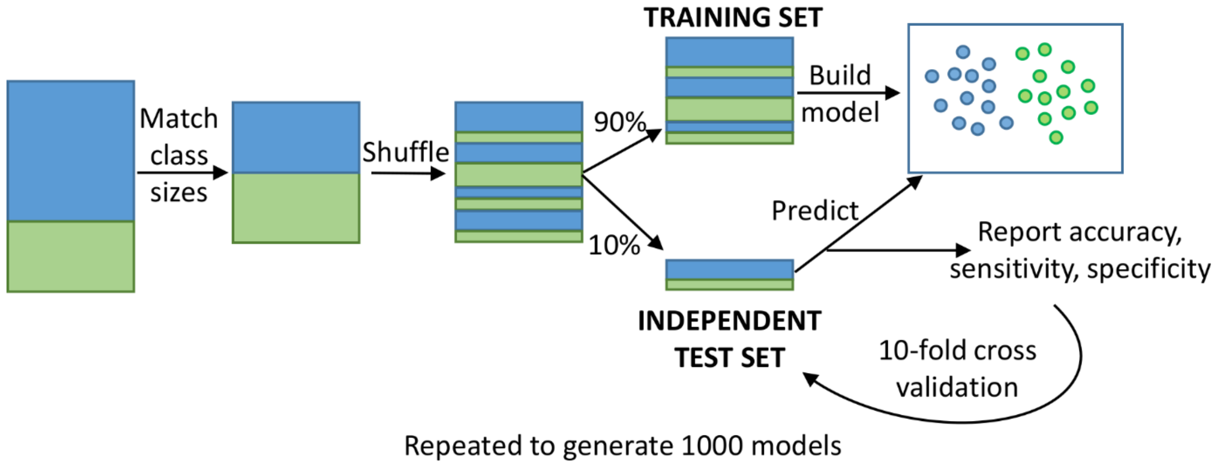


**Supplementary Fig. 1** Schematic representation of the statistical analysis scheme employed.

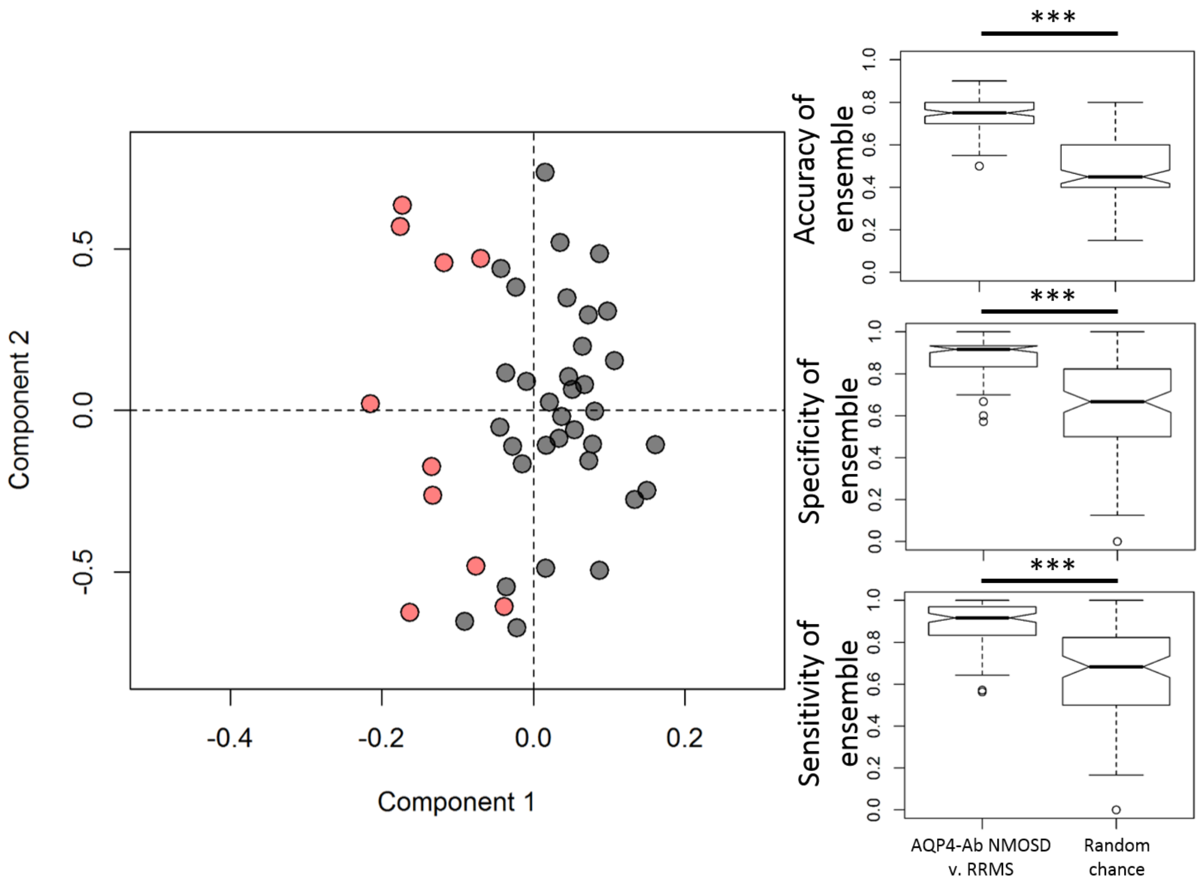


**Supplementary Fig. 2** A representative OPLS-DA scores plot of RRMS (black) and AQP4-Ab NMOSD (red) spectra from patients not treated with steroids. The accuracy, specificity, and sensitivity of the ensemble of 1000 models is significantly greater than random chance. P-values determined by the Kolmogorov-Smirnov test less than 0.001 are indicated by ***

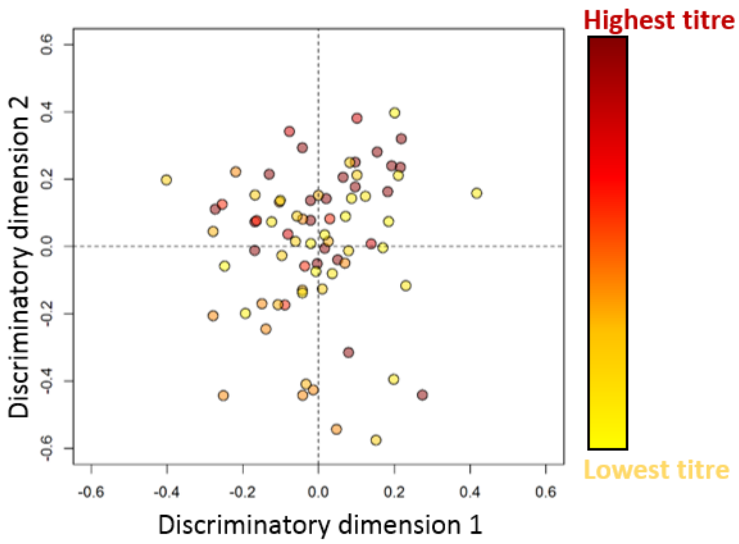


**Supplementary Fig. 3** A PCA scores plot of AQP4-Ab NMOSD plasma spectra. No clustering is observed as a result of antibody titre.

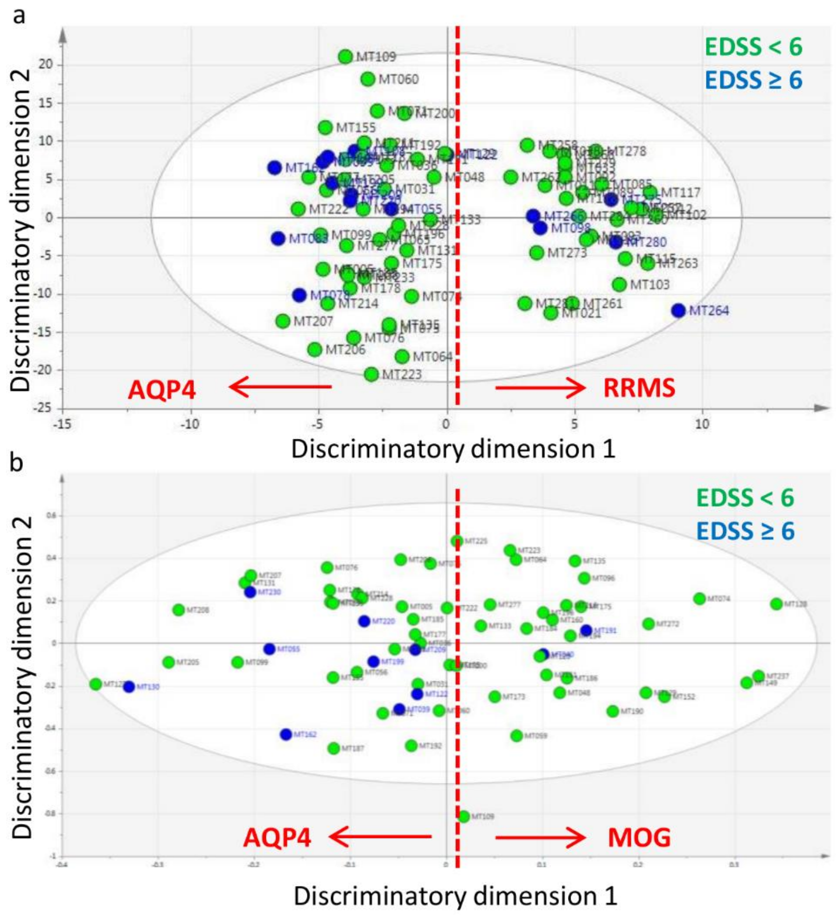


**Supplementary Fig. 4** OPLS-DA scores plots of AQP4-Ab NMOSD vs. (a) RRMS and (b) MOG-Ab disease. There is no clustering due to EDSS. High EDSS scores ≥ 6 (green) are spread throughout the groups with lower EDSS < 6 (blue)

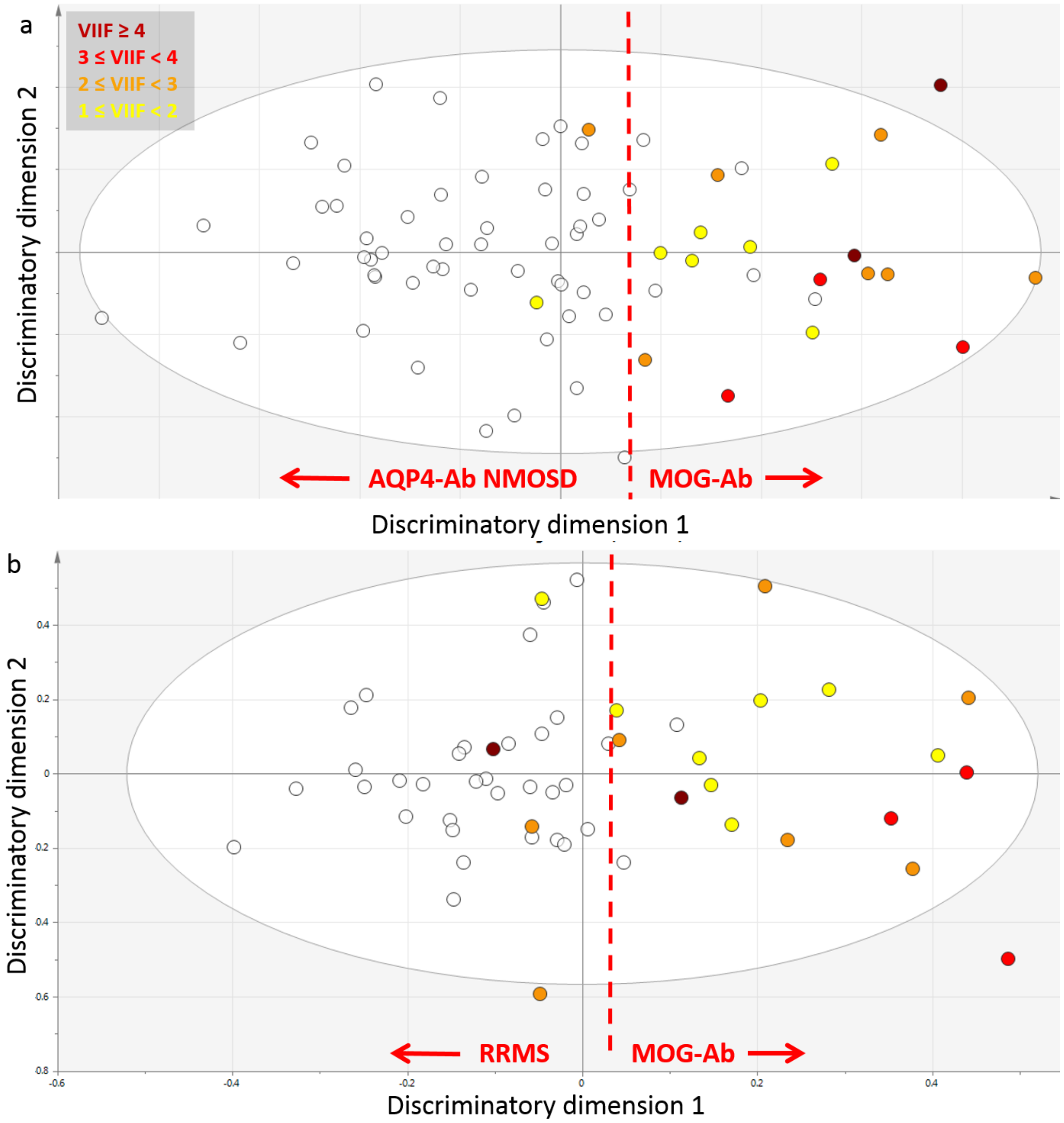


**Supplementary Fig. 5** OPLS-DA scores plots of MOG-Ab disease vs. (a) AQP4-Ab NMOSD and (b) RRMS. There is no clustering of the MOG-Ab samples due to the semi-quantitative MOG-ab visual immunofluorescence intensity scores (VIIS) at serum dilution 1:20. VIIS ≥ 4 (dark red), 3 ≤ VIIS < 4 (red), 2 ≤ VIIS < 3 (orange), and 1 ≤ VIIS < 2 (yellow) are spread throughout the MOG-Ab cluster. AQP4-Ab NMOSD and RRMS samples (unfilled circles) were all negative on the MOG-Ab assay.

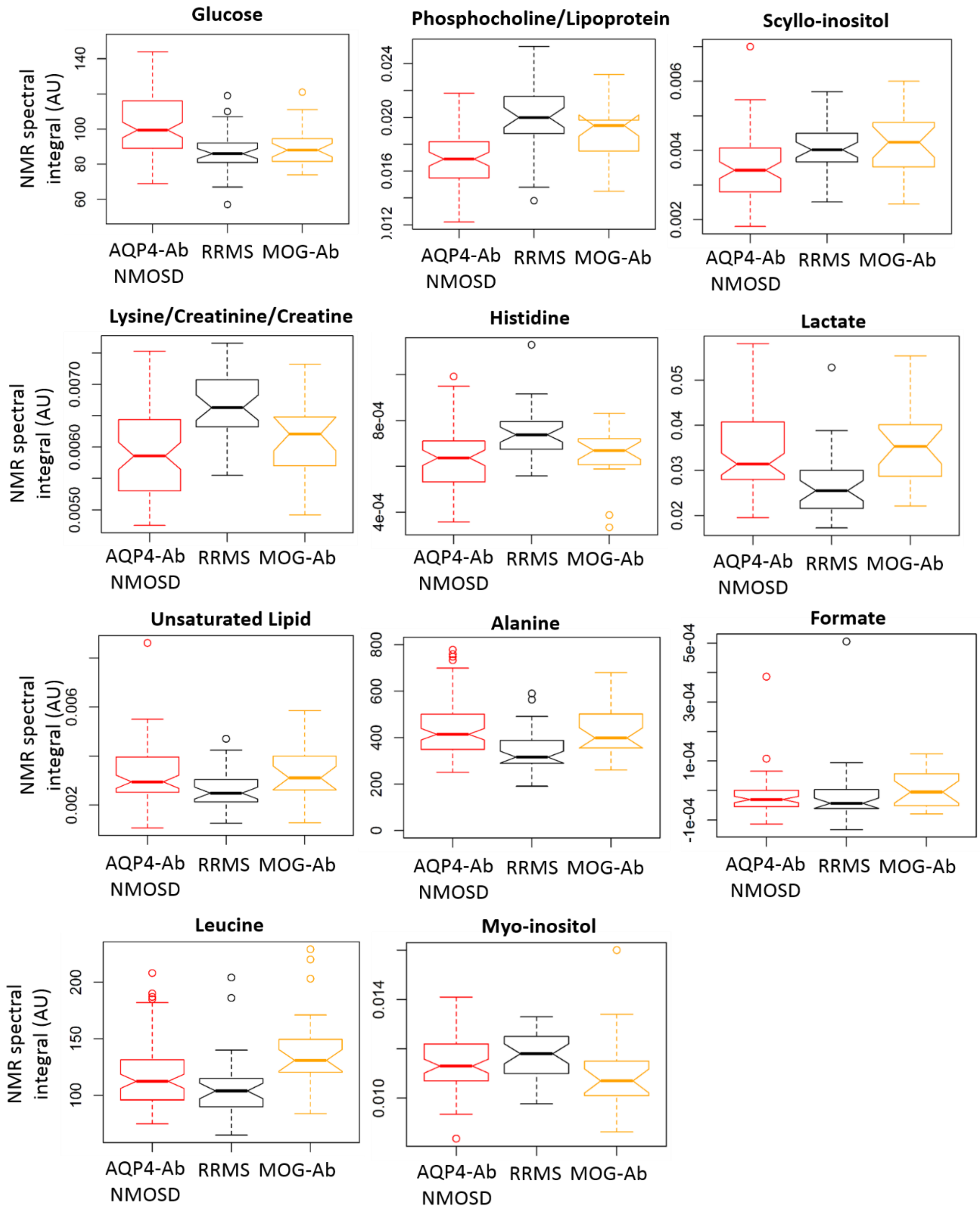


**Supplementary Fig. 6** Box plots of spectral integral values of each metabolite with significant changes across groups identified by multivariate analysis.
